# Directional charge separation in isolated organic semiconductor crystalline nanowires

**DOI:** 10.1038/ncomms10629

**Published:** 2016-02-25

**Authors:** J. A. Labastide, H. B. Thompson, S. R. Marques, N. S. Colella, A. L. Briseno, M. D. Barnes

**Affiliations:** 1 Department of Chemistry, University of Massachusetts, Amherst Massachusetts 01003, USA; 2 Department of Polymer Science and Engineering, University of Massachusetts, Amherst Massachusetts 01003, USA; 3 Department of Physics, University of Massachusetts, Amherst Massachusetts 01003, USA

## Abstract

One of the fundamental design paradigms in organic photovoltaic device engineering is based on the idea that charge separation is an extrinsically driven process requiring an interface for exciton fission. This idea has driven an enormous materials science engineering effort focused on construction of domain sizes commensurate with a nominal exciton diffusion length of order 10 nm. Here, we show that polarized optical excitation of isolated pristine crystalline nanowires of a small molecule n-type organic semiconductor, 7,8,15,16-tetraazaterrylene, generates a significant population of charge-separated polaron pairs along the *π*-stacking direction. Charge separation was signalled by pronounced power-law photoluminescence decay polarized along the same axis. In the transverse direction, we observed exponential decay associated with excitons localized on individual monomers. We propose that this effect derives from an intrinsic directional charge-transfer interaction that can ultimately be programmed by molecular packing geometry.

## Abstract (web summary)

Photo-induced charge separation in organic semiconductors usually occurs at interfaces between electron donors and acceptors. Here, the authors show using photoluminescence measurements that charge separation is intrinsic and directional in organic crystalline nanowires made of stacked monomers.

## Graphical abstract

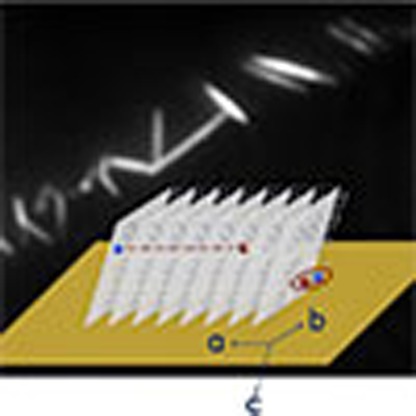

In the conventional view of the mechanistic operation of organic photovoltaic (OPV) devices, optically prepared Frenkel excitons migrate via diffusive transport (hopping) to a heterojunction where the interfacial energy drives exciton dissociation into polaron (electron and hole) pairs^1^,^2^,^3^. However, radiative recombination^3^,^4^,^5^,^6^,^7^,^8^, along with other quenching losses, contribute to a significant reduction in charge harvesting efficiency; a problem that has driven an enormous international research effort aimed at controlling donor and acceptor domain sizes to minimize such losses.

Diffusional transport of localized excitons in organic semiconductors is mediated by coupling between neighbouring chromophores, where the coupling mechanism is usually taken to be a Coulombic dipole–dipole (Frenkel exciton, FE) coupling^9^. Both the sign and magnitude of the matrix element, *V*_*ij*_, describing the nearest-neighbour coupling are important in aggregates of conjugated organic materials: the exciton diffusion coefficient scales as | *V*_*ij*_ |, while the sign of the interaction determines the energy ordering of coupled states (the lowest energy state for *V*_*ij*_ >0 contains *N-1* nodes, where *N* is the number of coupled chromophores, whereas the lowest energy state for *V*_*ij*_<0 is nodeless). This gives rise to the familiar H- or J- aggregate spectroscopic behaviour in vibronic structure in organic semiconductors^10^. Recently, Spano and Yamagata proposed that short-range charge-transfer (CT) coupling—augmenting the usual Coulombic dipole–dipole coupling mechanism—gives rise to hybrid HJ aggregate behaviour that includes both dipole–dipole and CT coupling^11^,^12^. Since CT interactions depend on wavefunction overlap between adjacent chromophores, the sign and magnitude of the interaction is highly sensitive to the precise packing geometry within the unit cell^13^,^14^. The combination of known crystal structure and high-level calculations of directional Frenkel exciton and CT couplings, along with experimental techniques for isolating individual crystalline nanowires, presents an interesting opportunity for investigation of directional coupling in small-molecule semiconductor assemblies.

A recently discovered n-type semiconductor of the rylene family of dyes, 7-8-15-16, tetraazaterrylene (TAT) readily forms high-quality, defect-free single (monoclinic) crystals by either physical vapour transport (PVT) or solution-phase self-assembly^15^,^16^. Figure 1 shows photoluminescence images of TAT nanowire crystals formed from self-assembly in toluene solution. Details of the crystal structure have been reported^15^, but briefly, the TAT molecules pack in a herringbone type arrangement in the *b*–*c* plane, and *π*-stacking occurs along the *a* axis. Several features of the TAT crystal structure are important to point out here. The molecular chromophore orientation is nearly orthogonal (∼80°) to the *π*-stacking axis. In addition, the chromophore arrangement in the *b*–*c* plane is such that dipole–dipole coupling is nearly zero, and the molecules are sufficiently separated spatially so that CT coupling is also very weak (approximately 5 meV for in-plane coupling, versus 150 meV along the *π*-stacking direction. Thus both dipole–dipole (Frenkel exciton) and CT coupling occur almost exclusively along the crystallographic *a* direction.

The combined effects of Frenkel exciton and CT coupling were illustrated recently in the quantitative spectral simulations by Yamagata and Spano^13^. Superficially, the absorption and photoluminescence spectra of isolated TAT extended crystals appear like a simple H-type aggregate, with 0–0 peak intensity diminished with respect to that of the 0–1 vibronic transition^10^. However, simulation of the absorption spectrum revealed a competition between a long-range H-type Frenkel exciton component, and a short-range J-type CT component, where interference between the two couplings results in J-like spectral features at low energies, and H-like spectral features at higher energies, ultimately yielding absorption (and photoluminescence) spectra with hybrid H/J character. Since the chromophore and *π*-stacking directions are approximately orthogonal (transverse and parallel to the crystal long axis respectively)^15^, absorption and emission polarized along the *π*-stacking axis must be mediated by the CT interaction, as dipole–dipole coupling preserves (transverse) polarization. We observed similar effects in isolated P3HT nanofibers where the interplay between CT and Frenkel exciton coupling interconverts inter- and intra-chain excitons at different rates depending on the degree of *π*-stacking registration between lamellar stacks.^17^

Here we demonstrate directional charge separation within pristine isolated single crystals of a small molecule n-type semiconductor TAT via manipulation of excitation polarization. Enabled by a strong charge-transfer coupling along specific orientations in the crystal^11^,^13^, pulsed illumination polarized along the *π*-stack direction (crystallographic *a* axis) forms short-lived inter-chromophore excitations that decompose into polaron pairs. This charge separation is signalled by power-law decay in the photoluminescence whose amplitude and decay exponent depend on the aspect ratio of the crystal. This study demonstrates the potential for both enhancement and directional control of photogenerated charge-separation efficiencies in small-molecule semiconductor assemblies without the need for an interface.

## Results

### Photoluminecence imaging of isolated nanowire crystals

**Figure 2:**
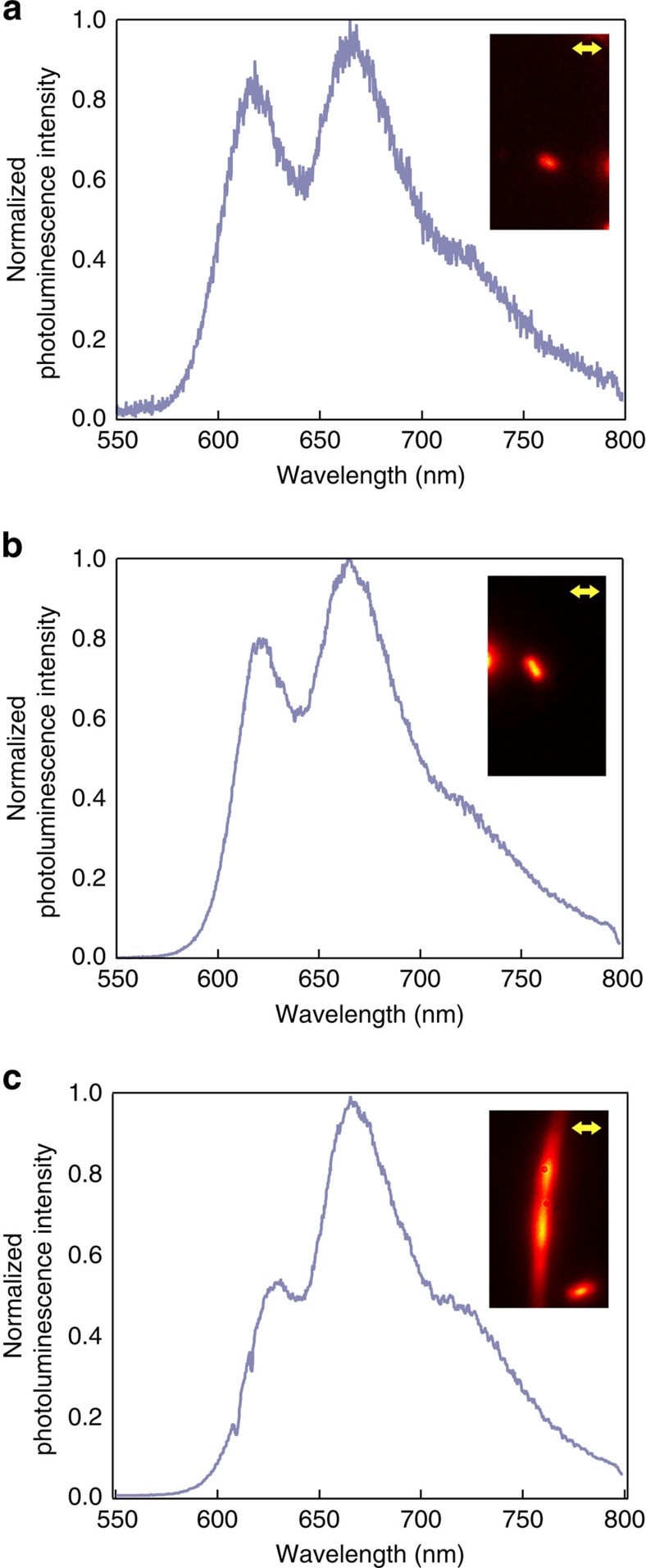
PL spectral evolution with increased crystal aspect ratio. PL spectra of (**a**) a nanocrystallite with an AR approximately equal to 1, (**b**) a crystal with an AR >1 and <3 and (**c**) a nanowire with an AR ≥3 with corresponding PL images, Scale bar, 300 nm. Note the qualitative changes in PL spectra indicating the structural envelope.

Figure 2 shows PL images along with corresponding emission spectra of different TAT assemblies formed from chloroform solutions cast on glass substrates, illustrating the spectral evolution of combined H/J coupling with increasing crystal size and presumed π-stacking order. Comparison of PL spectra from sampled solid-state assemblies with those from solution phase indicated that essentially all the aggregation is heavily seeded in solution, and promotes larger scale assembly during solvent evaporation. The different intensities in vibronic features in PL indicate that the dominant coupling depends on aggregation state; in the small (diffraction limited) nanocrystallite shown in Fig. 2a, the emission spectrum is characterized by an intensity ratio of vibronic components close to unity, while the corresponding intensity ratio for the extended crystal PL spectrum (Fig. 2c) is ∼0.5, and is essentially indistinguishable from those of physical vapour transport (PVT) grown crystals^16^. The origin of this spectral evolution will be discussed in detail elsewhere, but the spectral differences suggest different contributions of short-range charge-transfer and longer range Coulomb interactions, depending on the spatial extent of the crystal.

**Figure 3:**
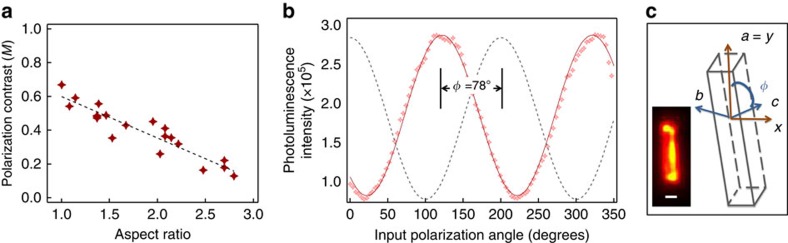
Polarization contrast in excitation with varying crystal aspect ratio. (**a**) Polarization contrast (*M*=(*I*_max_−*I*_min_)/(*I*_max_+*I*_min_)) for TAT crystallites of varying aspect ratios fit with a linear regression (dashed line). (**b**) The intensity trajectory over ∼2*π* rotation of excitation polarization, referenced to the crystal long axis (dashed line). (**c**) Chromophore orientation relative to the crystallographic *a* axis (long axis) as determined from the phase of the intensity modulation. Scale bar , 300 nm.

Figure 3 shows measured excitation polarization anisotropies (total PL intensity versus excitation polarization orientation) for crystals of varying aspect ratio (AR). Interestingly, the smallest nanocrystallites (AR≈1 as determined from the PL image) show the highest degree of polarization anisotropy (*M*≈0.6), while elongated crystals show only a modest polarization contrast (*M*≈0.2) in excitation. It is important to note that, in the absence of any interaction along the *π*-stacking direction (nominally orthogonal to the chromophore axis), the photoluminescence response to polarized excitation along this direction should approach zero. This also supports the picture of crystal size dependence of the relative CT and Frenkel exciton contributions to crystal absorption and PL, where the Frenkel exciton contribution increases with increasing crystal size and *π*-stacking order which increases the absorption strength along the *a* axis. The inset in Fig. 2 shows the variation of PL intensity with rotating excitation polarization, referenced to the crystal *a* axis (grey trace). The PL maximum occurs at *φ*=78±3°, in good agreement with the chromophore orientation determined from the crystal structure. It is important to point out that while smaller excitation anisotropy parameters are usually associated with disordered systems, the PL spectra of the large aspect ratio crystals (AR>3), are identical to those of pristine PVT-grown crystals, and therefore not associated with disorder.

### Time- and polarization-resolved photoluminescence imaging

**Figure 4:**
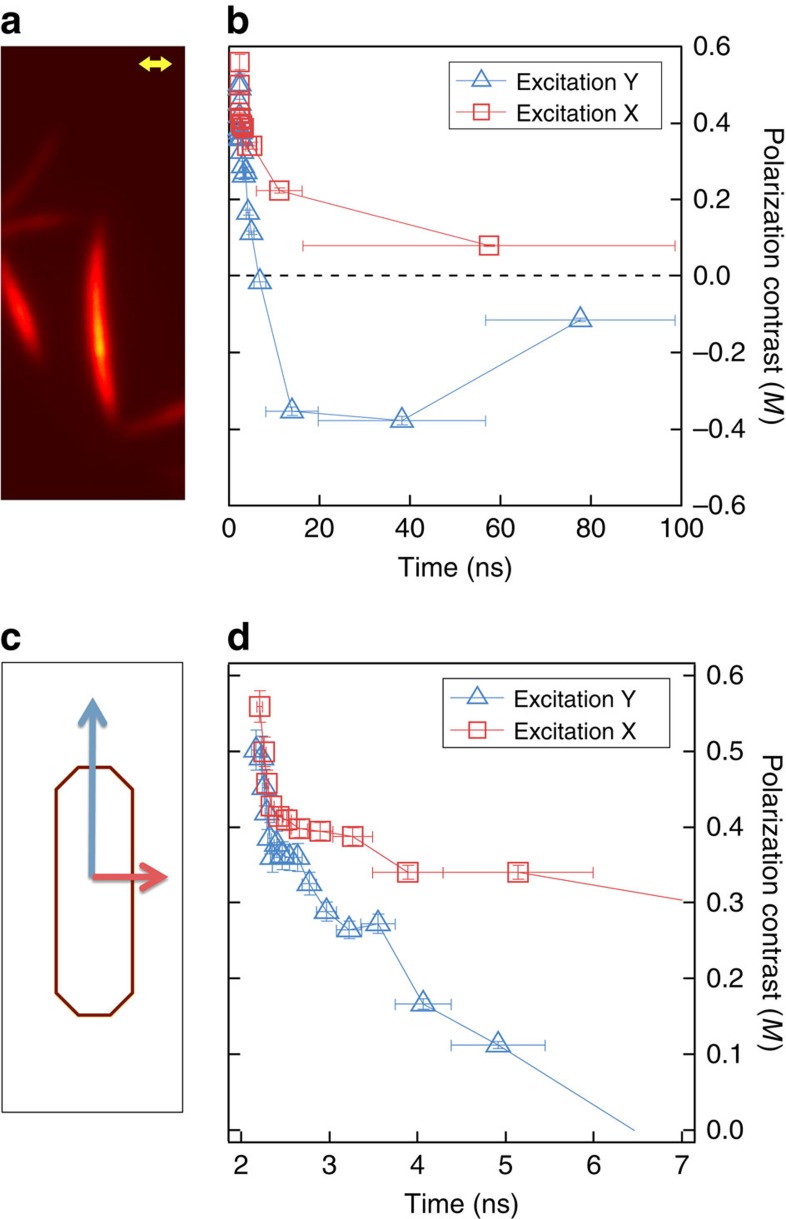
Time-resolved polarization contrast in photoluminescence for X- and Y-polarized light. (**a**) PL image of a TAT nanowire, Scale bar, 300 nm. (**b**) Time evolution of polarization contrast in PL for X-polarized (red squares) and Y-polarized (blue triangles) excitation. (**c**) Polarization detection scheme. (**d**) Short time behaviour of the polarization contrast for the different excitation polarizations. Vertical error bars for (**b**,**d**) represent 1 s.d. in polarization contrast for each point containing the same total number of counts, and horizontal error bars were determined by the averaged time in each step; thus nonlinear binning in time increases the horizontal uncertainty with increasing time.

Figure 4 shows the time evolution of the polarization contrast in photoluminescence from a TAT crystal for excitation polarized parallel (Y-polarized, blue triangles) and perpendicular (X-polarized, red squares) to the crystal *a* axis. Here, we defined time-dependent polarization contrast as *M*(*t*)*=*(*N*_X_(*t*)*−N*_Y_(*t*))/*(N*_X_(*t*)*+N*_Y_(*t*)), where *t* is the photon arrival time relative to the excitation pulse. In order to keep the uncertainty in *M*(*t*) approximately constant, we used a nonlinear binning such that the total number of counts was the same for each selected time step. Note that at early times (Fig. 4d, ∼80% of the photoluminescence is X-polarized (parallel to the chromophore axis) irrespective of excitation polarization, with only a small difference of *M*(*t*=0) of 0.58 for X-polarized excitation versus 0.5 for Y-polarized excitation. The polarization contrast in PL near *t*≈0 suggests that initially delocalized excitations relax on an ultrafast timescale to create Frenkel excitons, localized primarily individual molecules, and a relatively small fraction (∼25%) on adjacent *π*-stacked pairs. In both cases, the polarization contrast decreases rapidly (see inset detail) but depends sensitively on excitation polarization. For X-polarized excitation, the PL becomes almost completely depolarized after about 40 ns. For Y-polarized excitation, the effect is far more marked, showing an inversion of the sign of *M(t)* after ∼5 ns, indicating the formation of longer-lived species with transition moments polarized approximately along the *π*-stack axis.

## Discussion

**Figure 5:**
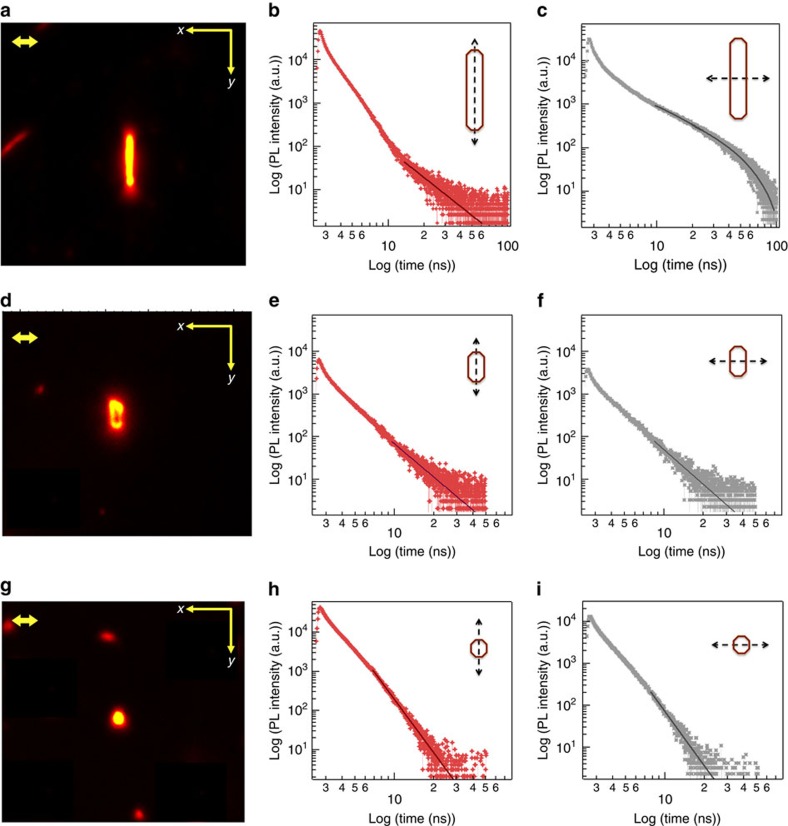
Time- and polarization-resolved PL decay dynamics with varying crystal aspect ratio. (**a**,**d**,**g**) PL images of TAT nanowire, crystal and nanocrystallite, respectively, Scale bar, 300 nm. (**b**,**c**,**e**,**f**,**h**,**i**) X- and Y-polarized PL decays from nanowire, crystal, and nanocrystallite for excitation along the *y* axis. For the nanowire (**a**), the Y-polarized PL (**b**) follows a power-law decay (*μ*=2.3), while the X-polarized PL decay (**c**) is bi-exponential (*γ*1=5.1 ns and *γ*2=20.2 ns). For the crystal (**d**), both polarization channels (**e**,**f**) show power-law decay with *μ*_*X*_=2.7 and *μ*_*Y*_=2.6. For the nanocrystallite (**g**), luminescence also follows a power-law decay in both polarization channels (**h**,**i**) with *μ*_*X*_≈*μ*_*Y*_=4.5.

The mechanistic origin of this behaviour was revealed by looking at the PL decay in each polarization channel in detail. Figure 5 shows the PL images from a nanowire (Fig. 5a), crystal (Fig. 5d) and nanocrystallite (Fig. 5g), along with PL decay decomposed into X- and Y-polarization components with excitation polarized along the Y- direction (parallel to the *π*-stack axis). At short times, the PL follows an exponential decay in both channels characteristic of excitonic emission. Interestingly, at long times, the PL decay dynamics of the nanowire are determined by the excitation polarization; the Y-polarized luminescence (Fig. 5b) follows a power-law decay (*I*(*t*)*≈A·t*^*−*(*1+μ*)^, where *A* is an amplitude, and *μ* is the power-law exponent) with *μ*=1.3, while the X-polarized PL decay (Fig. 5c) is exponential. In contrast, the PL decay from the crystal, being thicker in the transverse dimension and having a smaller aspect ratio than the nanowire, follows a power-law in both X and Y polarization channels with *μ*_X_=1.7 and *μ*_Y_=1.6. For the diffraction limited nanocrystallite, the PL decay follows a power-law in both X and Y polarization channels, but with much larger characteristic decay constants (*μ*_X_*≈μ*_Y_=3.5).

As demonstrated by Silva and coworkers^3^, power-law decay in photoluminescence from assemblies of organic semiconductors is the signature of two-body polaron pair recombination, with amplitudes and exponents reporting on the efficiency of polaron generation and characteristic timescales for their recombination. In semicrystalline P3HT films, Silva and coworkers showed that exciton regeneration occurs via tunnelling of geminate charge-pairs at amorphous-crystalline interfaces, thus concluding that the mechanism for charge separation in P3HT was extrinsic, requiring an interface between amorphous and crystalline domains to drive exciton dissociation. This was corroborated by recent work by Silva and Stingelin^18^, which highlighted the role of mixed domains, as well as work on isolated crystalline P3HT nanofibres^17^ (which show only very small power-law components) and semicrystalline P3HT nanoparticles^19^, which show very robust power-law photoluminescence. The question here is whether the delayed photoluminescence in TAT crystals is derived from exciton regeneration by tunnelling (similar to P3HT) or through interaction of mobile carriers within the crystal.

Polaron-pairs can be formed either by unimolecular exciton fission or via bi-molecular triplet–triplet interactions. In pentacene for example, the very low-lying triplet energy (0.86 eV) enables efficient conversion of singlet excitons into two triplets, which then undergo annihilation to form charge pairs^20^,^21^,^22^. Like P3HT films, the delayed PL in TAT crystals scales linearly with pump intensity corresponding to exciton formation via charge-pair recombination with distributed kinetics, thus ruling out bi-molecular triplet–triplet annihilation, which would be expected to show a nonlinear dependence on pump intensity. The question remains whether the exciton regeneration mechanism in TAT crystals occurs via tunnelling between more-or-less immobile charge-pairs similar to the mechanism in P3HT, or two-body encounters between mobile carriers. Estimates of carrier diffusion distance based on computed electron/hole hopping energies in TAT crystals (50 and 4.5 meV for electron and hole, respectively) suggest that anisotropic carrier mobility on the experimental time scale should significantly contribute the polaron pair recombination kinetics.^23^

**Figure 6:**
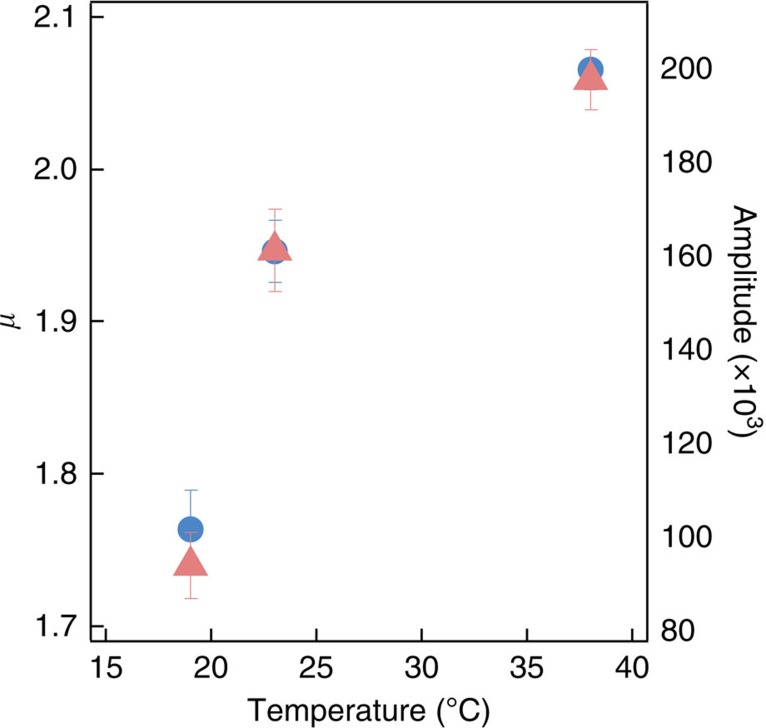
Temperature dependence of power-law exponent and amplitude. Power-law exponent *μ* (blue circles) and amplitude (red triangles) recovered from fits to delayed photoluminescence from the same TAT crystal for Y-polarized excitation and emission as a function of temperature, with s.d. error bars. Both follow an approximate *T*^1/2^ dependence.

To address this issue, we looked at the temperature dependence of the delayed photoluminescence. Figure 6 shows the temperature dependence of both amplitude and power-law exponent in Y-polarized luminescence (with Y-polarized excitation) obtained from the same TAT nanocrystal. In contrast with the results by Silva and coworkers^3^, we find that both amplitude and exponent scale approximately like *T*^1/2^ clearly indicating that exciton regeneration is not associated with tunnelling, but rather diffusion of carriers along the *a* axis. This suggests a physical picture in which exciton regeneration occurs via encounter of diffusing carriers within a certain capture radius. While more work is required to quantitatively identify the temperature dependence, the physical picture appears to be analogous to bi-molecular reactive kinetics, with an increase of regenerated excitons per unit time, which appears in our measurement as increased amplitude of the power-law signal. Note that if the initial charge-separation process was thermally activated, we should expect to see nonlinear (exponential) increase in amplitude with increasing temperature. Within the same analogy, we can interpret the temperature dependence in the power-law exponent as a decrease in the mean radius resulting in faster exciton regeneration and photoluminescence decay.

In the small, diffraction limited nanocrystallites, we observed large, polarization–isotropic power-law exponents and relatively early power-law onset (*t*<7 ns). Here the short-range charge-transfer interaction dominates inter-chromophore coupling, and promotes the geminate formation and efficient dissociation of both inter and intra-chromophore excitations. The larger power-law exponents are indicative of smaller average electron-hole radius, likely due to lower mobilities associated with *π*-stacking disorder in the smaller assemblies. The increase in *π*-stacking order in the extended crystals shifts the balance of interactions towards Coulomb coupling, reducing the number of geminate polarons formed, and the rate of Frenkel exciton dissociation, but also increases the diffusive mobility along the *a* axis. The combination of these factors leads to later power-law onsets, smaller amplitudes, and factor of 2 smaller exponents seen in the power-law decay from extended crystals. In these crystals, the mean electron-hole distances calculated from the combination of experimentally determined power-law exponents and calculated zero field mobilities are too large for recombination via tunnelling. We propose that exciton dissociation and diffusion-mediated polaron-pair recombination, rather than geminate polaron pairs that recombine via tunnelling, is responsible for the power-law signal polarized in the *b*–*c* plane seen from the large crystals.

A fascinating directionality of the PL decay mechanism in isolated TAT crystals was revealed using time- and polarization-resolved photoluminescence spectroscopy. For the crystalline nanowires studied, excitation polarized along the *π*-stacking axis results in PL polarized along the same axis that decays according to a power-law with exponent whose magnitude depends on crystal aspect ratio, while the PL polarized transverse to the crystallographic *a* axis (approximately aligned along the chromophore axis) follows an exponential decay characteristic of Frenkel excitons localized on individual molecular sites. For thicker crystals with smaller aspect ratios, power-law decays characteristic of polaron pair recombination was seen for PL polarized in all directions, perhaps indicating a minimum spatial extent required for non-geminate polaron pair generation in the lateral dimension. In contrast with P3HT films, for example, the mechanism for charge-pair formation in TAT crystals appears to be intrinsic, deriving from a strong charge-transfer coupling along the *π*-stacking axis. These results illustrate the potential of harnessing directional charge-transfer interactions in crystalline molecular assemblies for enhanced charge-separation without the need for diffusion and interfacial dissociation. While interfacial engineering efforts within the field have generated invaluable insights to mitigate associated efficiency losses, not having to rely on an interface to generate charge-separated states is of considerable advantage. We have shown that by simple manipulation of the crystal aspect ratio and the excitation polarization, it is possible to directly access charge-transfer interactions that could offer a significant advantage to improving power conversion efficiencies in organic photovoltaic devices. Further, the ability to make macroscopically directionally ordered films of TAT single crystals has been well demonstrated, and represents a platform with which we can exert directional control over energy and charge flow in small-molecule semiconductor assemblies. Finally, it is important to point out that the observations reported here are not unique to TAT; the design principles that dictate direction and magnitude of Coulomb and charge-transfer interactions apply to a broad class of small molecule and oligomeric semiconductors materials whose packing geometry can be tuned by molecular architecture.

## Methods

### Experimental methodology

We employed a variant of time-tagged time-resolved (T^3^R) photon counting^24^,^25^ called time- and polarization-resolved photoluminescence^17^,^19^ to probe directional coupling in isolated TAT crystals. Photons emitted from the sample are sorted into two orthogonal polarization components detected by avalanche photodiodes whose outputs are processed through a high-speed router, thus identifying the arrival time and polarization state of each individual detected photon^17^,^19^, enabling direct measurement of the time-evolution of polarization contrast with resolution ultimately limited by the time-to-digital converter (4 ps in our implementation). This technique is particularly well suited for probing polarization evolution mechanisms in crystalline assemblies because the orientation of the crystal long axis in the lab frame is easily discernable from the photoluminescence image, thus defining an orientation with respect to the excitation polarization. Because the molecular packing structure is known, the photoluminescence in different polarization channels can be referenced to either the molecular chromophore orientation or *π*-stacking direction.

### Sample preparation

Isolated TAT nanowires and nanocrystallites were prepared by sonication of a 0.5 μM TAT in chloroform solution for roughly 1 h. A small amount of solution was then drop-cast onto a plasma cleaned glass coverslip resulting in well-dispersed nanowires and nanocrystallites with a density of ∼10 crystals per 100 μm^2^. The solution was left for 1–2 days for subsequent aggregation, allowing for the formation of decreased aspect ratio crystals. After 1–2 days, a small amount of aggregated solution was drop-cast onto a plasma cleaned glass coverslip with ∼5 crystals per 100 μm^2^.

### Optical measurements

The time- and polarization- resolved photoluminescence measurements were done using a home built microscope described by ref. 17. Isolated nanostructures were excited with a pulsed diode laser (485 nm) with an average input intensity of approximately 16 W cm^−2^, a pulse width (FWHM) of 90 ps, and a repetition rate of 10 MHz. The PL spectra were taken on a separate microscope set-up with a continuous-wave excitation using an air-cooled argon ion laser (488 nm).

## Additional information

**How to cite this article:** Labastide, J. A. *et al.* Directional charge separation in isolated organic semiconductor crystalline nanowires. *Nat. Commun.* 7:10629 doi: 10.1038/ncomms10629 (2016).

## Figures and Tables

**Figure 1:**
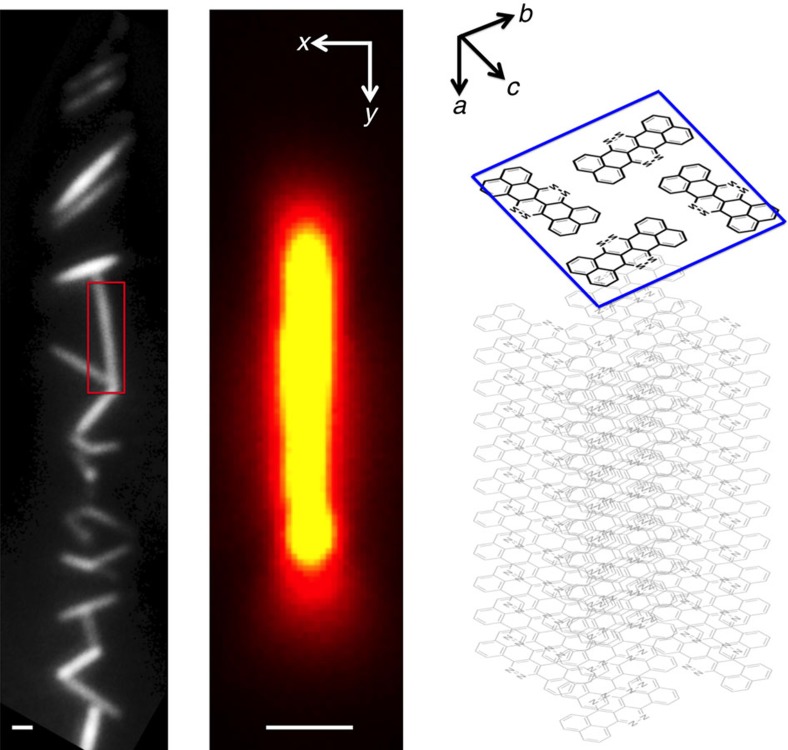
Orientation and structural schematic of TAT nanowire crystals. (Left) Photoluminescence (PL) of TAT nanowire crystals formed from solution-phase self assembly. (Center) PL image of isolated TAT nanowire with *π*-stacking direction oriented along laboratory *Y* axis. (Right) Structural schematic of TAT nanowire crystal showing relative orientation of crystallographic axes {*a*, *b*, *c*} with respect to laboratory *X*–*Y* plane. Scale bar, 300 nm.
